# Fibroblast Activation Protein-Targeted Photodynamic Therapy of Cancer-Associated Fibroblasts in Murine Models for Pancreatic Ductal Adenocarcinoma

**DOI:** 10.1021/acs.molpharmaceut.3c00453

**Published:** 2023-07-24

**Authors:** Daphne N. Dorst, Esther M. M. Smeets, Christian Klein, Cathelijne Frielink, Daan Geijs, Marija Trajkovic-Arsic, Phyllis F. Y. Cheung, Martijn W. J. Stommel, Martin Gotthardt, Jens T. Siveke, Erik H. J. G. Aarntzen, Sanne A. M. van Lith

**Affiliations:** † Department of Medical Imaging, Radboud University Medical Center, 6525 GA Nijmegen, The Netherlands; ‡ Roche Pharma Research and Early Development, Innovation Center Zurich, 8952 Schlieren, Switzerland; § Department of Pathology, Radboud University Medical Center, 6525 GA Nijmegen, The Netherlands; ∥ Bridge Institute of Experimental Tumour Therapy, West German Cancer Center, University Hospital Essen, University of Duisburg-Essen, 47057 Essen, Germany; ⊥ Division of Solid Tumour Translational Oncology, German Cancer Consortium (DKTK Partner Site Essen) and German Cancer Research Center, DKFZ, 69120 Heidelberg, Germany; # Department of Surgery, Radboud University Medical Center, 6525 GA Nijmegen, The Netherlands

**Keywords:** fibroblast activation protein (FAP), cancer-associated fibroblast (CAF), targeted photodynamic therapy (tPDT), pancreatic ductal adenocarcinoma (PDAC), syngeneic models

## Abstract

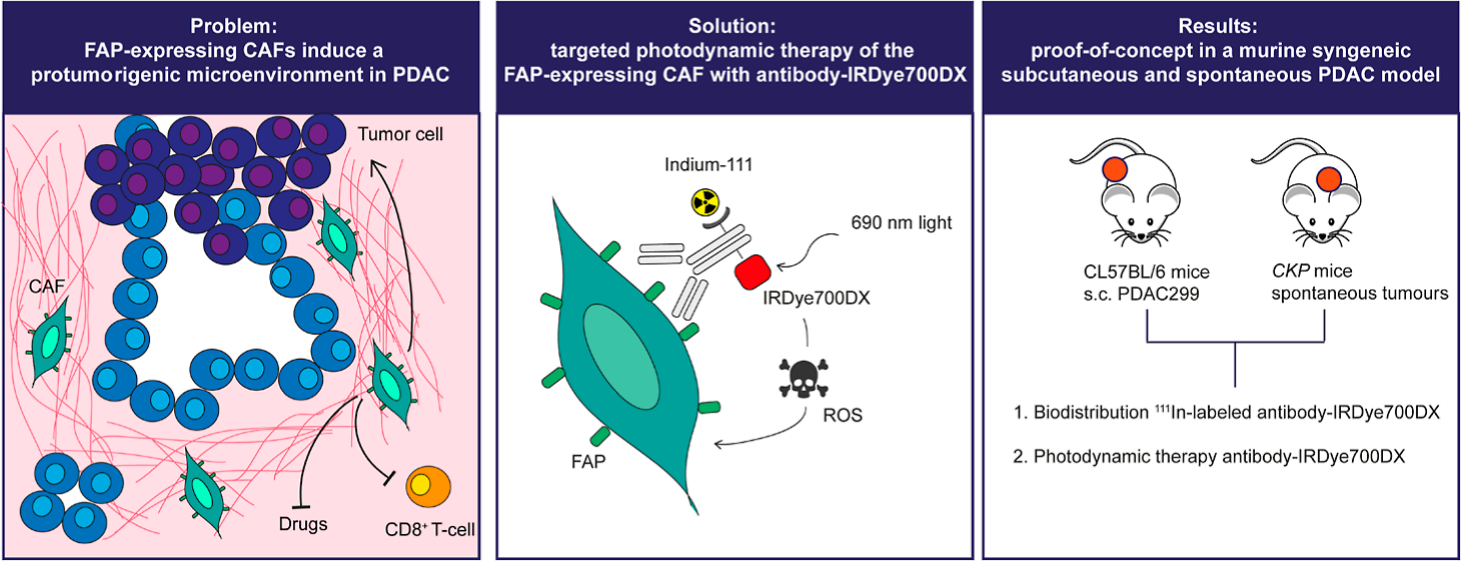

Patients with pancreatic ductal adenocarcinoma (PDAC) have a dismal 5 year survival of 9%. One important limiting factor for treatment efficacy is the dense tumor-supporting stroma. The cancer-associated fibroblasts in this stroma deposit excessive amounts of extracellular matrix components and anti-inflammatory mediators, which hampers the efficacy of chemo- and immunotherapies. Systemic depletion of all activated fibroblasts is, however, not feasible nor desirable and therefore a local approach should be pursued. Here, we provide a proof-of-principle of using fibroblast activation protein (FAP)-targeted photodynamic therapy (tPDT) to treat PDAC. FAP-targeting antibody 28H1 and irrelevant control antibody DP47GS were conjugated to the photosensitizer IRDye700DX (700DX) and the chelator diethylenetriaminepentaacetic acid. In vitro binding and cytotoxicity were evaluated using the fibroblast cell-line NIH-3T3 stably transfected with FAP. Biodistribution of ^111^In-labeled antibody-700DX constructs was determined in mice carrying syngeneic tumors of the murine PDAC cell line PDAC299, and in a genetically engineered PDAC mouse model (*CKP*). Then, tPDT was performed by exposing the subcutaneous or the spontaneous PDAC tumors to 690 nm light. Induction of apoptosis after treatment was assessed using automated analyses of immunohistochemistry for cleaved caspase-3. 28H1-700DX effectively bound to 3T3-FAP cells and induced cytotoxicity upon exposure to 690 nm light, whereas no binding or cytotoxic effects were observed for DP47GS-700DX. Although both 28H1-700DX and DP47GS-700DX accumulated in subcutaneous PDAC299 tumors, autoradiography demonstrated that only 28H1-700DX reached the tumor core. On the contrary, control antibody DP47GS-700DX was only present at the tumor rim. In *CKP* mice, both antibodies accumulated in the tumor, but tumor-to-blood ratios of 28H1-700DX were higher than that of the control. Notably, in vivo FAP-tPDT caused upregulation of cleaved caspase-3 staining in both subcutaneous and in spontaneous tumors. In conclusion, we have shown that tPDT is a feasible approach for local depletion of FAP-expressing stromal cells in murine models for PDAC.

## Introduction

Patients with pancreatic ductal adenocarcinoma (PDAC) have a poor prognosis with a 5 year overall survival of 9%.^1^ In PDAC tumors, up to 90% of the mass can be composed of tumor stroma, consisting of different components including blood vessels, immune cells and cancer-associated fibroblasts (CAFs). The latter secrete extracellular matrix components, including collagen fibrils and hyaluronic acid.^2^ This results in high interstitial pressure and limited perfusion and thereby hampers penetration of chemotherapeutics and lymphocyte infiltration.^3,4^ Besides, CAFs secrete chemokines which decrease tumor T-cell infiltration.^5^ All of these factors may contribute to the low success rates of chemo- and immunotherapies in PDAC.^6,7^

Several studies have shown that the depletion of the abundant tumor stroma resulted in an increased sensitivity of the tumor tissue to traditional chemotherapeutic agents, as well as enhanced immune cell infiltration in preclinical models.^8−10^ Depleting myofibroblasts in mice with spontaneous PDAC tumors using a conditional knockout of alpha-smooth muscle actin; however, was associated with increased metastatic spread, impaired immune response to the tumor and decreased survival.^11^ Therefore, depletion of all CAFs is not a feasible approach. Instead targeting specific, pathological, subsets is a more attractive option.

One marker that gained increased attention the past years is fibroblast activation protein (FAP).^12^ FAP is a dipeptidyl peptidase abundantly expressed in fibroblasts involved in wound healing,^8^ and its expression has been linked to CAFs in a host of different solid malignancies, including PDAC.^13^ Researchers developed pharmacological FAP inhibitors,^14^ FAP-targeting vaccines and T-cell therapies,^15−18^ or FAP-targeting molecules coupled to directly toxic compounds, such as therapeutic radionuclides or toxins.^19−23^ Though these compounds show efficacy in various preclinical tumor models, and augment anti-tumor immune responses, in some studies, severe systemic toxicity was found, including cachexia and anaemia.^24,25^ This reflects the importance of FAP-expressing cells in maintaining normal muscle mass and haematopoiesis and warrants caution because of FAP-expression in wound healing and normal tissues such as the placenta, uterine stroma, embryonic tissue, and multipotent bone marrow stromal cells.^10,26,27^

Targeted photodynamic therapy (tPDT) offers a potential solution to the abovementioned limitations. In tPDT, a photosensitizer is conjugated to a targeting compound of interest. Upon activation with light of a specific wavelength, the photosensitizer induces the formation of reactive oxygen species (ROS), which cause damage to the cells to which the conjugate is bound. Since the conjugate is inert without light activation, this allows for systemic administration and subsequent local activation by light.

Here, we investigated the feasibility of using tPDT for the depletion of FAP-expressing CAFs. We determined the biodistribution and light-induced cytotoxicity of a FAP-targeting monoclonal antibody conjugated to the phthalocyanine photosensitizer IRDye700DX in mice with subcutaneous syngeneic PDAC tumors and in *CKP* mice spontaneously developing PDAC tumors.

## Experimental Section

### Cell Culture

NIH-3T3 fibroblasts stably transfected with murine FAP (3T3-FAP; PETR4906) were cultured in DMEM supplemented with 10% FCS, penicillin, streptomycin, and 1.5 μg/mL puromycin at 37 °C in a humidified atmosphere with 5% CO_2_. PDAC299 cells (derived from a pancreatic tumor of a Ptf1a^WT/Cre^ Kras^WT/LSL–G12D^;p53^LSLR172H/fl^ mouse^28^) were cultured in DMEM containing glutaMAX and pyruvate supplemented with 10% FCS at 37 °C in a humidified atmosphere with 5% CO_2._

### Antibody Conjugation and Characterization

Monoclonal IgG1 antibody 28H1 (having high affinity for murine (<1 pM) and human (268 pM) FAP), and isotype control DP47GS (no known binding specificity and no affinity for murine or human FAP) were used. Both antibodies have P329G LALA mutations in the Fc domain,^29^ which abolishes FcyR and C1q binding and thus immune effector functions. 28H1 and DP47GS were conjugated to *N*-hydroxysuccinimide IRDye700DX (NHS-IRDye700DX, Licor, Lincoln, USA) and *p*-isothiocyanatobenzyl DTPA (ITC-DTPA, Macrocyclics, Plano, USA) using a molar ratio of 1:6:10 for antibody/NHS-IRDye700DX/ITC-DTPA, as described previously.^30^ This resulted in the conjugates DTPA-28H1-IRDye700DX and DTPA-DP47GS-IRDye700DX, which will now be abbreviated as 28H1-700DX and DP47GS-700DX throughout the paper. UV–visible absorbance spectra of the two conjugates, 28H1-700DX and DP47GS-700DX, were recorded at 300–800 nm in PBS with the Tecan Infinite 200 Pro (Tecan, Mannedörf, Switzerland). Emission spectra at 650–800 nm were recorded after 620 nm excitation.

### Singlet Oxygen Production

28H1-700DX or DP47GS-700DX (250 nM) were incubated with *p*-nitrosodimethylaniline (RNO; 50 μM) and imidazole (400 μM) in PBS in clear flat-bottom 96-wells plates. They were irradiated with 690 nm light at 290 mW/cm^2^, using a LED device (LEDfactory, Leeuwarden, The Netherlands).^31^ Absorbance at 440 nm was measured every minute with the Tecan Infinite 200 Pro to determine ^1^O_2_-induced bleaching of RNO.

### Radiolabeling with Indium-111 and Quality Control

28H1-700DX and DP47GS-700DX were incubated with 0.2 Mbq/μg [^111^In]InCl3 (Curium, Petten, The Netherlands) and twice the volume of 0.5 M 2-(*N*-morpholino)ethanesulfonic (MES) buffer, pH 5.5, for 30 min at room temperature (RT). Labeling efficiency and radiochemical purity were determined by instant thin-layer chromatography (ITLC) on a silica-gel chromatography strip (Biodex, Shirley, NY, USA) using 0.1 M citrate buffer pH 6.0 as the mobile phase.

### In Vitro Binding and Internalization

3T3-FAP cells were detached with 10 mM EDTA and incubated with 1600 Bq ^111^In-labeled 28H1-700DX or DP47GS-700DX in binding buffer (DMEM with 0.5% w/v BSA) on ice or at 37 °C for 2 h in suspension. After washing twice with PBS, cells were collected and activity was counted in a γ-counter (2480 Wizard 3″, LKB/Wallace, Perkin-Elmer, Boston, MA, USA).

### In Vitro tPDT

3T3-FAP cells were plated in 96-wells plates and grown until 80% confluency. Cells were incubated with either 28H1-700DX or DP47GS-700DX in concentrations ranging from 0.037 nM to 3 nM in binding buffer, or with binding buffer alone, at 37 °C in a humidified atmosphere with 5% CO_2_ for 2 h. After washing with PBS, regular culture medium was added. The cells were then exposed to 50 J/cm^2^ 690 nm light using the LED light source at a fluency rate of 290 mW/cm^2^. Cell viability was measured 24 h after tPDT using the CellTiter-Glo assay (Promega, Madison, USA).

### Animal Studies

The Radboud University animal ethics committee approved all study protocols (CCD number AVD103002015209 and AVD1030020209645). Procedures were performed according to the Institute of Laboratory Animal Research guide for Laboratory Animals.

Subcutaneous tumor model—6–8 weeks old female C57/BL6 mice (6JRj, Janvier) were fed standard chow *ad libitum* and housed on a 12 h day–night cycle. After acclimatization for 1 week upon arrival at our institution, they were injected subcutaneously at one or both shoulders with 0.5 million PDAC299 tumor cells in 100 μL DMEM. Mice were weighed and tumors were measured with a caliper twice a week. When (one of the) tumors reached a size of 200 mm^3^, the mice were included in the experiments.

Spontaneous tumor model—male and female *Ptf1a*^*wt/Cre*^*;Kras*^*wt/LSL-G12D*^*;p53*^*fl/fl*^ (*CKP*) mice (C57BL/6 background)^32^ were included in the experiments at approximately 6 weeks of age. In these experiments, we used both male and female mice, as the number of mice from breeding was limited. An overview of the number of mice and their characteristics is given in Table S1.

### Ex Vivo Biodistribution

Mice carrying subcutaneous PDAC299 tumors (*N* = 5–6/group) or *CKP* mice with spontaneous pancreatic tumors (*N* = 3–4/group) were injected intravenously with 50 μg antibody-700DX labeled with 1 MBq (ex vivo biodistribution only) or 10 MBq (SPECT/CT scans and subsequent ex vivo biodistribution) ^111^In in 200 μL of PBS. Three additional mice with subcutaneous PDAC299 tumors were co-injected with an 10× excess protein dose of unlabeled 28H1-700DX to determine FAP-specificity of the uptake. Mice were euthanized by CO_2_/O_2_ suffocation at either 48 or 96 h post injection for the subcutaneous model and at 24 h post injection for the *CKP*. Tissues of interest were resected and weighed, and tissue uptake of ^111^In was measured using a γ-counter (WIZARD, 2480 Automatic Gamma Counter, Perkin-Elmer, Boston, MA, USA) and calculated as the percentage of the injected activity per gram of tissue (% IA/g). An overview of included animals is given in Table S1.

### SPECT/CT

SPECT/CT scans were acquired for either 1 h at 24 or 48 h post injection or for 1.5 h at 96 h post injection using a U-SPECT/CT-II (MILabs, Utrecht, The Netherlands). Images were acquired using a 1 mm diameter pinhole ultrahigh sensitivity mouse collimator or a 1 mm diameter pinhole rat collimator (*n* = 1). SPECT scans were followed by CT scans (65 kV, 615 μA). All SPECT scans were reconstructed with 3 iterations and 16 subsets and a voxel size of 0.4 mm (MILabs reconstruction software). SPECT images were made with VivoQuant software. The tissues from the mice that underwent the SPECT/CT scans were also used for the ex vivo biodistribution as described above after scanning.

### Ex Vivo Autoradiography

Half of each subcutaneous PDAC299 tumor was snap frozen in liquid nitrogen, and they were cut into 4 μm sections and mounted on superfrost glass slides. A phosphor screen (Fuji Film BAS-IP SR 2025, Raytest, Straubenhardt, Germany) was exposed to the slides for 72 h in a Fujifilm BAS cassette 2025. Then, the images were acquired with a Typhoon FLA 7000 laser scanner (GE healthcare Life Sciences, Chicago, IL, USA) at a pixel size of 25 × 25 μm. Images were analyzed with Aida Image analyser software (Raytest). Slides were stored at −80 °C until they were used for immunostainings.

Half of the *CKP* tumors was formalin-fixed and paraffin embedded (FFPE) immediately after resection. They were cut into 4 μm sections and mounted on superfrost glass slides. Autoradiography images were obtained as described above. The slides were stored at RT until they were used for immunostaining.

### In Vivo tPDT

Female mice carrying subcutaneous PDAC299 tumors were injected with 50 μg of 28H1-700DX in 200 μL of PBS (*N* = 6), DP47GS-700DX in 200 μL of PBS (*N* = 5) or PBS alone (*N* = 5). At 24 h post-injection, mice were anaesthetized with isoflurane (1.5% in 1 L/min oxygen). Uptake in both tumors was visualized with fluorescence imaging, excitation filter 640 nm, emission filter Cy5.5 (IVIS Lumina II, PerkinElmer, Waltham, MA, USA), and then, one of the tumors was exposed to 100 J/cm^2^ 690 nm light at 230 mW/cm^2^ using the LED device. We irradiated the left tumor, unless no tumor had grown at that side. The rest of the body was shielded from light using paper towels and aluminum foil. After light irradiation, the treated tumor was imaged again. Mice were sacrificed at 24 h post irradiation with CO_2_/O_2_ asphyxiation. Tumors were obtained and Formalin-Fixed and Paraffin-Embedded (FFPE) for immunohistochemical analyses.

Additionally, in vivo photodynamic therapy was performed in five *CKP* mice (four male, one female). Mice were injected intravenously with 50 μg of 28H1-700DX (*N* = 2) or DP47GS-700DX (*N* = 3) in 200 μL of PBS. Twenty four hours after injection, mice were anaesthetized with isoflurane anesthesia (1.5% in 1 L/min oxygen), and tumors were exposed by making an excision in the abdominal wall. Other internal organs and the rest of the body were shielded from light using tissues and aluminum foil. The tumor was subsequently exposed to 52 J/cm^2^ 690 nm light at 290 mW/cm^2^ using a LED.^31^ Only a part of the tissue was exposed to light. The part that was left inside the body was shielded from light and served as internal non-irradiated control. The mice were kept under anaesthesia, on a heated pad to prevent hypothermia, for 45 min to 1 h after light irradiation (see Table S1), and subsequently sacrificed by cervical dislocation. The tumor and organs of interest were isolated and remaining fluorescence was visualized with fluorescence imaging, excitation filter 640 nm, emission filter Cy5.5, 1 min acquisition using the IVIS system. The tumor tissue was subsequently FFPE processed for immunohistochemical analyses. An overview of the included mice is given in Table S1.

### Immunohistochemistry

FFPE tumors were sectioned at 5 μm thickness. Sections were stained with hematoxylin and eosin (H&E) for visualization of the morphology, and subsequently, the spontaneous *CKP* tumors were graded by the G-score system by MTA. This grading system was used to include the mice that have a G-stage of at least 1 in our subsequent analyses.

Immunohistochemistry staining of cleaved caspase-3 (9661S, Cell signalling technologies, 1/4000) was performed. The slides were deparaffinized by xylene wash and rehydrated using ethanol. Antigen was retrieved using 10 mM citrate (pH 6.0) for 10 min at 96 °C. The peroxidase activity was blocked by incubating with 3% H_2_O_2_ in PBS. Non-specific binding was blocked through preincubation with 20% NGS for 30 min. Next the slides were incubated with the primary cleaved caspase-3 antibody (Cell signaling technologies, 9661S, 1/4000 in PBS/1% BSA) at 4 °C overnight. Slides were incubated with the secondary biotinylated goat anti-rabbit antibody (VECTASTAIN, Thermo-Fisher, 1/200 in PBS/1% BSA), followed by labeling with the avidin-biotin complex (VECTASTAIN, Thermo-Fisher, 1/100). The antibody complex was visualized using diaminobenzene (bright DAB, Immunologic). All slides were counterstained with hematoxylin and mounted with a cover slip (Permount, Thermo-Fisher).

Frozen sections used for autoradiography were stored at −80 °C before staining with rabbit-anti-FAP (EPR20021, ab 207178, Abcam, binds to both human and murine FAP). Slides were air-dried for 2 h at RT, fixed with ice-cold acetone for 10 min and air-dried again for 2 h at RT. Endogenous peroxidase activity was blocked with 3% H_2_O_2_ in PBS for 10 min at RT in the dark, and endogenous biotin was blocked with the Biotin-Avidin blocking kit (Vector Laboratories) for 30 min. Upon incubation with 20% normal goat serum in PBS for 30 min at RT, sections were incubated with rabbit-anti-FAP (Abcam, 1:25 in PBS/1% BSA) for 1 h at RT. Subsequently sections were incubated with goat-anti-rabbit-biotin (Vector Laboratories, 1/100 in PBS/1% BSA) for 30 min at RT, and biotin/streptavidin ABC-elite complex for 30 min at RT in the dark. Signal was visualized with bright DAB (Immunologic) for 8 min at RT in the dark, and all slides were counterstained with hematoxylin and mounted with Permount.

### Immunohistochemistry Automated Quantification

Slide digitization was performed using a 3DHistech P1000 scanner digital slide scanner (3DHistech, Budapest, Hungary) with a 20× objective at a resolution of 0.24 μm/pixel. To quantify the amount of caspase-3 after FAP-tPDT, regions were manually annotated. For the subcutaneous PDAC299 tumors, we annotated the total tumor area on tissue sections from two different depths in the tumor (∼150 μm distance). For the spontaneous *CKP* tumors, we annotated the tumor area from the part exposed to light, and from the part not exposed to light on tissue sections from two different depths in the tumor (∼150 μm distance). Tissue folds and staining artifacts were excluded from the annotation. An automated color unmixing algorithm^33^ was used to unmix the DAB staining and the background haematoxylin staining. In short, this algorithm aims to find the optimal stain matrix per slide to ensure the best color unmixing. This unmixing is performed using an adapted version of the deconvolution method described in ref (34). We extended this algorithm by binarizing the unmixed stain concentrations using Otsu thresholding, to quantify the amount of staining present per unit area (resolution of 2 μm/pixel). The ratio was calculated by dividing the stain of interest by the total amount of stained pixels.

### Statistics

The statistical analyses were performed using GraphPad Prism 5.0 software. Quantitative data (SO production, cellular binding, cell death, and uptake in ex vivo biodistribution) are expressed as mean ± SD. Statistical significance was tested with a Mann–Whitney *U* test for comparison of two groups (in biodistribution analyses) and with a Kruskal–Wallis test for comparison of multiple groups (in caspase-3 upregulation analyses).

## Results

### 28H1-700DX Induces Cell Death of FAP-Expressing Cells In Vitro

**Figure 1:**
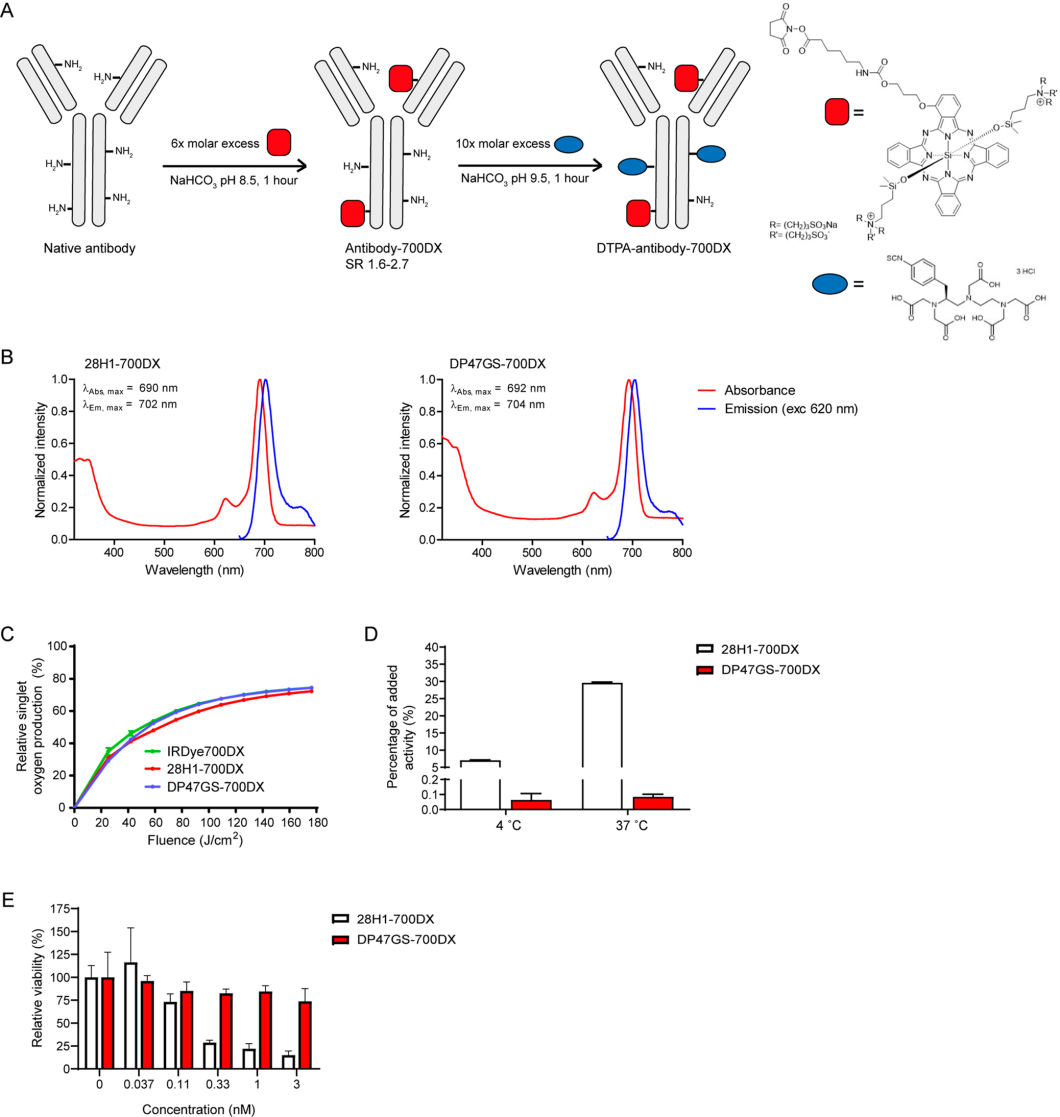
(A) Scheme for conjugation of NHS-IRDye700DX and ITC-DTPA to the antibodies 28H1 and DP47GS to generate DTPA-antibody-700DX, (B) absorbance and emission spectrum of 28H1-700DX and DP47GS-700DX at 620 nm excitation, (C) bleaching of RNO absorbance induced by singlet oxygen formation upon exposure to 280 mW/cm^2^ 690 nm light, (D) 3T3-FAP associated 28H1-700DX and DP47GS-700DX upon incubation on ice or at 37 °C, and (E) cell viability of 3T3-FAP upon incubation with 28H1-700DX or DP47GS-700DX and exposure to 50 J/cm^2^ 280 mW/cm^2^ 690 nm light.

The anti-FAP antibody 28H1 and control antibody DP47GS were conjugated to ITC-DTPA and NHS-IRDye700DX, reaching substitution ratios of the photosensitizer of 1.6–2.7 (Figure 1A). Spectral properties of IRDye700DX were retained after conjugation to both 28H1 and DP47GS (Figure 1B). Both 28H1-700DX and DP47GS-700DX induced the production of singlet oxygen with equal efficiency upon exposure to 690 nm light and at similar levels as the parent compound IRDye700DX (Figure 1C). Binding of the ^111^In-labeled conjugates to FAP-expressing 3T3 cells was observed only for 28H1-700DX and not for DP47GS-700DX (Figure 1D). Increased cellular association after 37 °C incubation when compared to incubation on ice, indicates that the majority of the antibody is internalized, when assuming that endocytosis is inhibited at low temperatures. Upon incubation with 28H1-700DX and DP47GS-700DX and exposure to 690 nm light, only 28H1-700DX induced protein-dose and light-dose dependent cell death of 3T3-FAP cells (Figure 1E). Importantly, in previous work, we have shown that 28H1-700DX does not induce toxicity of native 3T3 cells upon illumination, confirming FAP-specificity.^30^

### 28H1-700DX Accumulates into Subcutaneous PDAC299 Tumors

**Figure 2:**
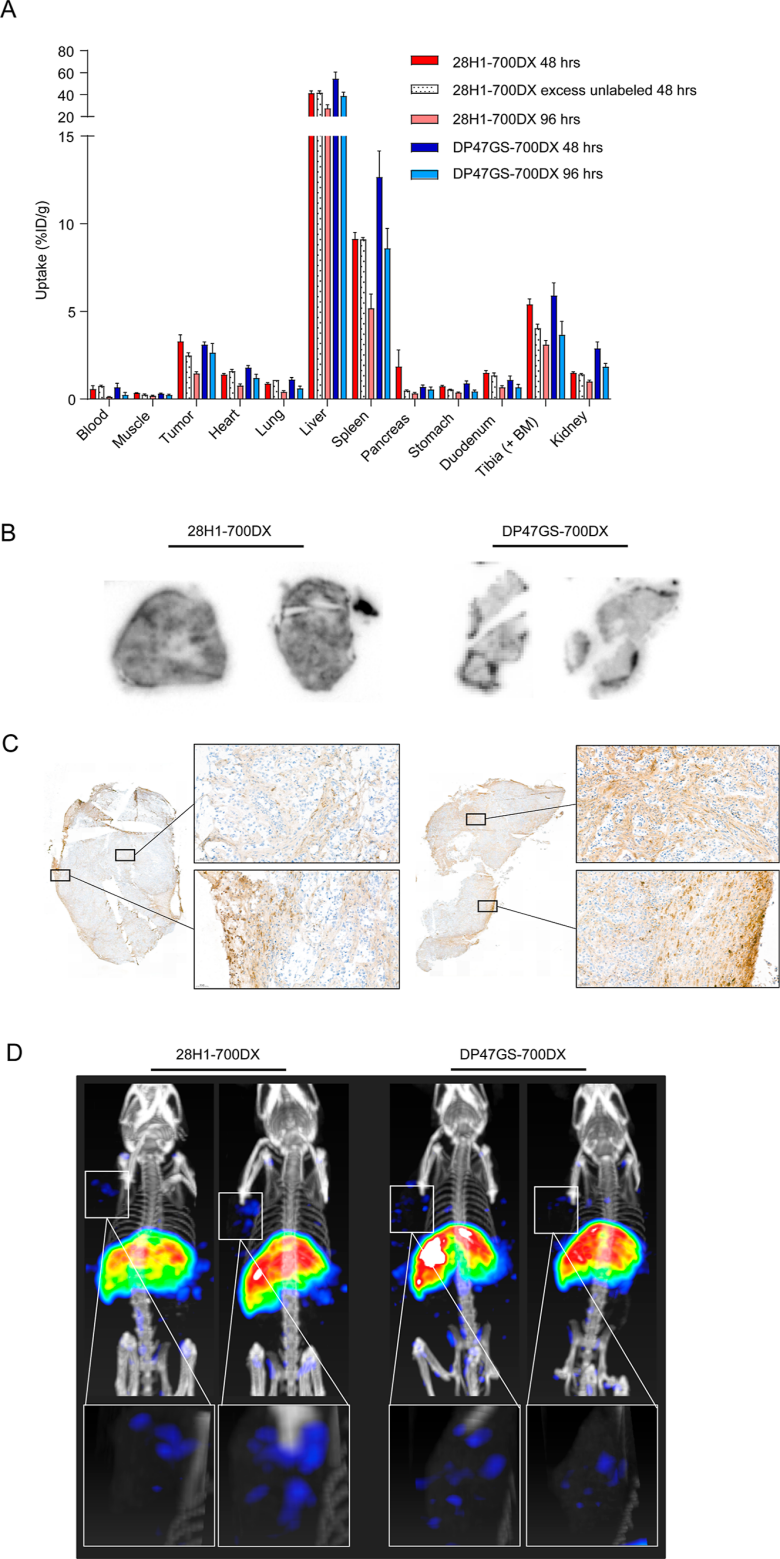
(A) Biodistribution of ^111^In-labeled 28H1-700DX or DP47GS-700DX in mice carrying subcutaneous PDAC299 tumors, (B) autoradiography of PDAC299 tumor sections at 48 h post injection of ^111^In-labeled 28H1-700DX or DP47GS-700DX, (C) FAP immunohistochemistry of PDAC299 tumor sections used for autoradiography, and (D) SPECT/CT images at 48 h post injection of ^111^In-labeled 28H1-700DX or DP47GS-700DX.

In mice carrying subcutaneous PDAC299 tumors, a relative tumor uptake of ^111^In-labeled 28H1-700DX of 3.30 ± 0.89 % IA/g and 1.47 ± 0.21 % IA/g was observed at 48 and 96 h post injection, respectively (Figure 2A and Table S2). Tumor uptake at 48 h was reduced upon co-injection of 10× excess non-labeled 28H1-700DX (2.49 ± 0.27 % IA/g, NS *p* = 0.095). Similar relative tumor uptake of ^111^In-labeled DP47GS-700DX was found after 48 h post injection (3.10 ± 0.36 % IA/g) and uptake was better retained after 96 h post injection (2.65 ± 1.17 % IA/g). High liver uptake of both antibodies was found, and moderate uptake in the spleen and tibia containing the bone marrow. Only the latter could be blocked with excess non-labeled 28H1-700DX, indicating FAP-specificity of uptake (5.41 ± 0.75 vs 4.05 ± 0.38, *p* = 0.048). Autoradiography of PDAC299 tumor sections showed heterogeneous distribution of ^111^In-labeled 28H1-700DX in the whole tumor, while very strong focal uptake of ^111^In-labeled DP47GS-700DX was mainly found in the periphery of the tumor at both 48 h post injection (Figure 2B) and 96 h post injection (Figure S1A). FAP-immunohistochemistry of these same tissue sections showed moderate staining of FAP-expressing cells throughout the whole tumor, which appeared to be in stromal regions, and strong staining of cells of unknown origin in the tumor borders (Figure 2C). SPECT/CT imaging visualized uptake in the liver, and low uptake of especially 28H1-700DX in the tumor region at 48 h only (Figure 2D). At 96 h post injection, only the liver was visualized on the SPECT/CT scan (Figure S1B).

### 28H1-700DX Accumulates into Tumors of CKP Mice

**Figure 3:**
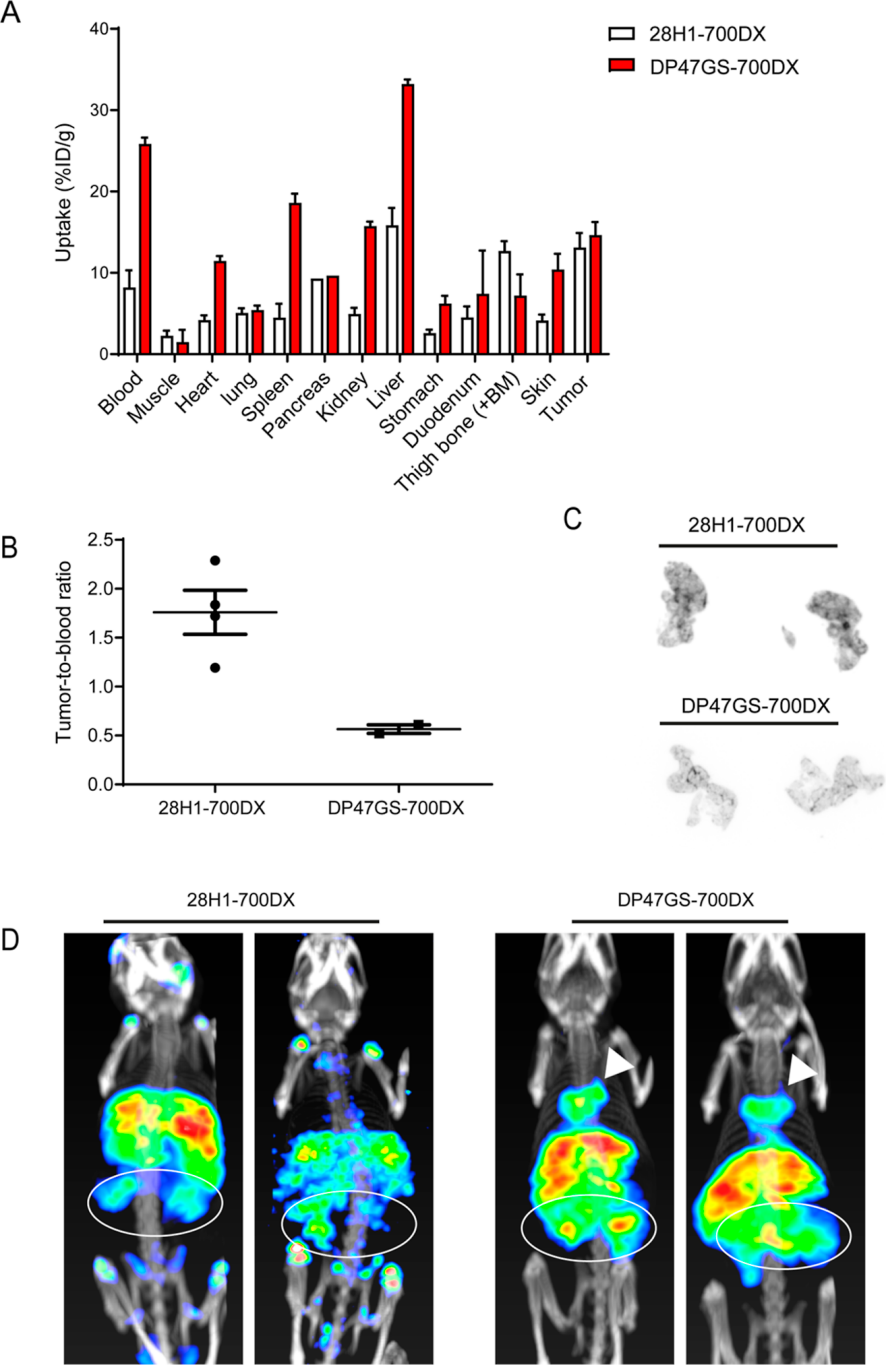
(A) Biodistribution of ^111^In-labeled 28H1-700DX or DP47GS-700DX in *CKP* mice at 24 h post injection, (B) tumor-to-blood ratios, (C) autoradiography of tumor sections at 24 h post injection of ^111^In-labeled 28H1-700DX or DP47GS-700DX, and (D) SPECT/CT scans at 24 h post injection of ^111^In-labeled 28H1-700DX or DP47GS-700DX. Note the uptake in the tumor region (circles) and the high uptake in the heart for the DP47GS-700DX (arrowheads).

Biodistribution of both ^111^In-labeled constructs was determined in *CKP* mice with spontaneous pancreatic tumors. One of the mice injected with ^111^In-labeled DP47GS-700DX was excluded from analyses, as extremely high lung uptake was found without a known cause (Figure S2). The biodistribution of ^111^In-labeled 28H1-700DX and ^111^In-labeled DP47GS-700DX in the other mice showed a comparable accumulation of both conjugates in the tumor (13.12 ± 3.55 vs 14.66 ± 2.25 % IA/g, respectively) at 23 ± 1 h post injection (Figure 3A and Table S3). Residual blood levels of ^111^In-labeled DP47GS-700DX were approximately three-fold higher compared to ^111^In-labeled 28H1-700DX (25.85 ± 1.10 % IA/g vs 8.22 ± 4.17, respectively). The tumor-to-blood ratio is therefore also higher in mice injected with ^111^In-labeled 28H1-700DX compared to ^111^In-labeled DP47GS-700DX (1.76 ± 0.45 vs 0.57 ± 0.06, respectively) (Figure 3B). Tracer accumulation in other organs such as liver, spleen, and kidney is also higher for DP47GS-700DX compared to 28H1-700DX, possibly as a consequence of the higher residual blood levels. Autoradiography revealed uptake of both antibodies with a heterogeneous uptake pattern (Figure 3C). SPECT/CT imaging confirmed these findings with clear visualization of the tumor and liver with both tracers (Figure 3D). The irrelevant antibody also showed uptake in the heart, representing the higher residual blood levels. Tracer accumulation in off-target organs is similar to that previously described in healthy C57BL/6 mice and mice with arthritis.^30^

### 28H1-700DX tPDT Induces Apoptosis in Subcutaneous PDAC299 Tumors

**Figure 4:**
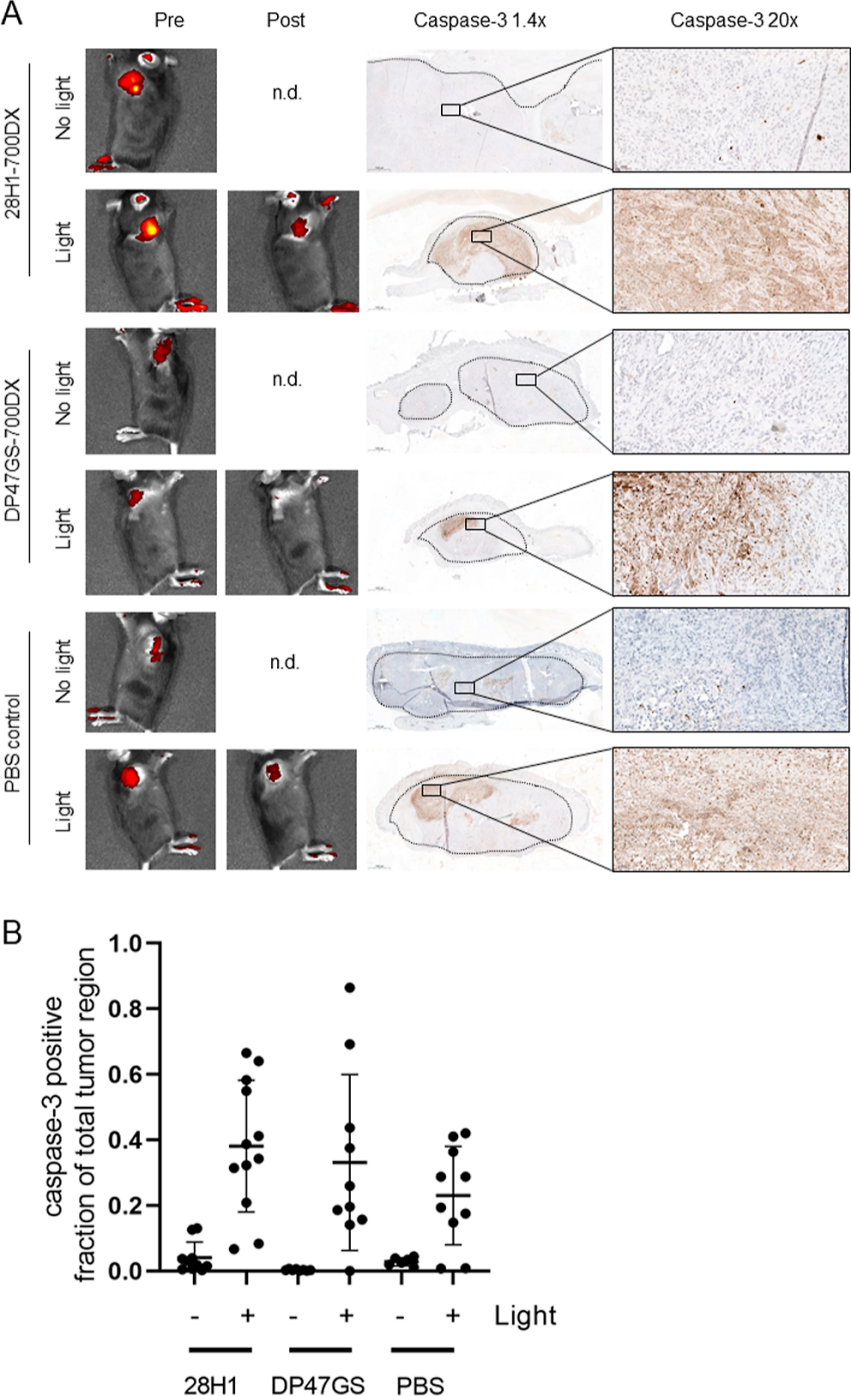
(A) Representative images of fluorescence imaging of mice carrying PDAC299 tumors before and after light exposure, and cleaved caspase-3 immunohistochemistry of tumors at 24 h after light exposure. All images are shown in Figures S3–S5. (B) Automated quantification of the cleaved caspase-3 positive fraction of the total tumor region in images originating from two different depths in one tumor.

To investigate FAP-PDT efficiency in a subcutaneous mouse model, we injected 50 μg of 28H1-700DX (*N* = 6) or DP47GS-700DX (*N* = 5) and PBS (*N* = 5) as a control. One of the tumors was exposed to 100 J/cm^2^ 690 nm light at 230 mW/cm^2^. Increased fluorescent signal in the tumors of 28H1-injected mice was observed when compared to the other groups, and bleaching of IRDye700DX-derived fluorescence after exposure to light indicated efficient activation of the photosensitizer (Figures 4A and S3–S5). The expression of cleaved-caspase-3 staining was analyzed in the manually annotated tumor regions by dividing the number of pixels positive for caspase-3 through the total number of pixels. Upregulation of expression was found in the irradiated tumors of all groups when compared to the tumors that were not exposed to light (Figures 4B and S3–S5). Though the caspase-3 positive fraction of the total tumor region was higher in the group treated with 28H1-700DX (0.42 ± 0.24), this was not significantly different when compared to the DP47GS-700DX or PBS-treated groups (0.33 ± 0.27 and 0.23 ± 0.15, respectively).

### 28H1-700DX tPDT Induces Apoptosis in Tumors of CKP Mice

**Figure 5:**
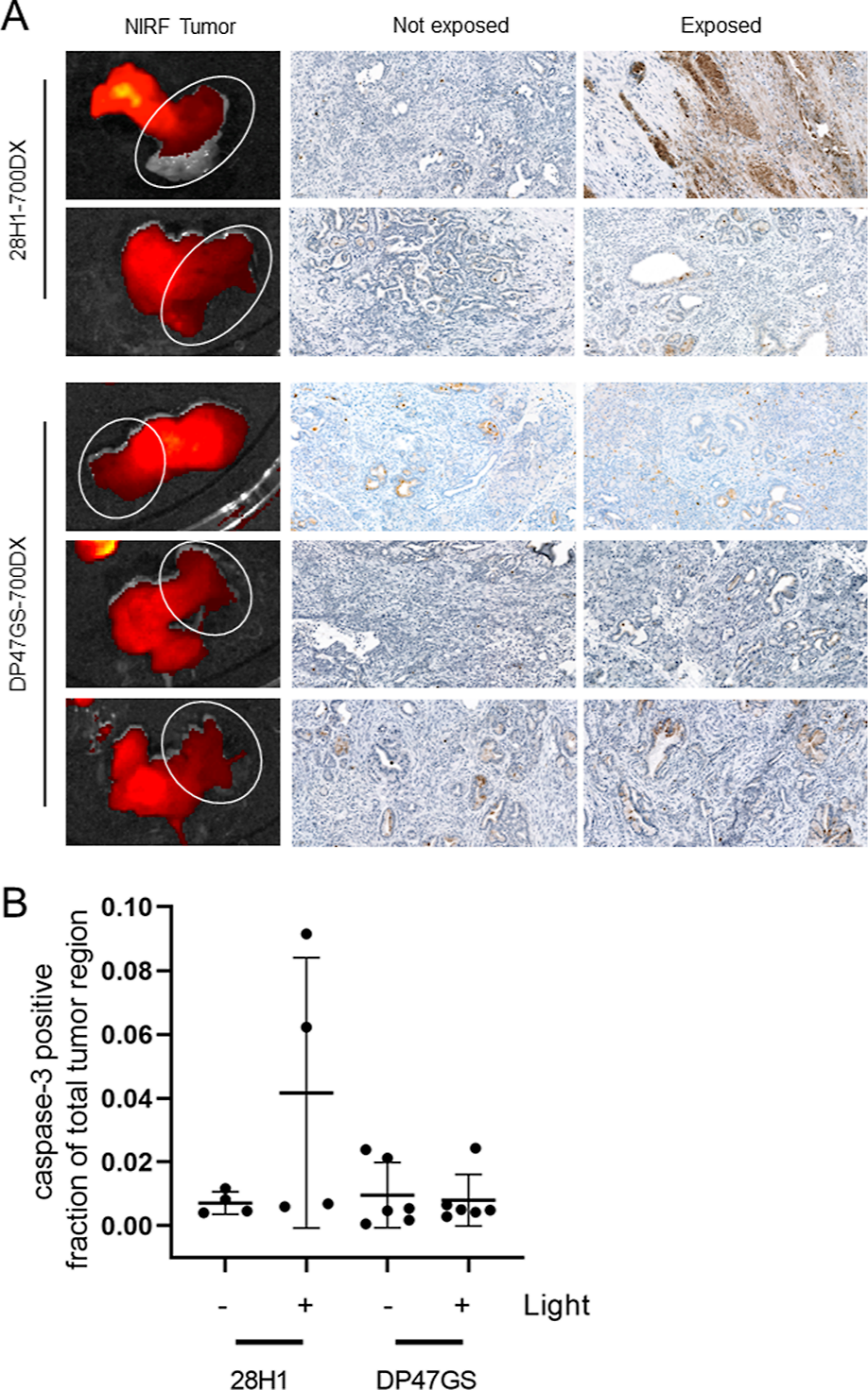
(A) Fluorescent images of *CKP* tumors after light exposure, and caspase-3 immunohistochemistry of these tumors. (B) Automated quantification of the cleaved caspase-3 positive fraction of the total tumor region in images originating from two different depths in one tumor.

tPDT was performed using 28H1-700DX (*N* = 2) and DP47GS-700DX (*N* = 3) conjugates at 23 ± 1 h post injection. Tumors were exposed by surgical incision of the abdomen, and irradiated with 52 J/cm^2^ 690 nm light at 290 mW/cm^2^. Only a part of the tumor was exposed to light, which resulted in an internal non-exposed control. Ex vivo fluorescence imaging showed quenching of the IRDye700DX signal predominantly in the part of the tumor that was exposed to light for both antibodies (Figure 5A). The caspase-3 staining was quantified in annotated regions in tumor tissue that were or were not exposed to light. Upon injection of 28H1-700DX and subsequent light exposure, we observed an increase in caspase-3 positive fraction of the annotated tumor region for 1 of the 2 mice, while no increase in caspase-3 positive fraction was observed in the non-irradiated internal controls and mice injected with DP47GS-700DX and subsequent light exposure (Figure 5B).

## Discussion

In this study, we investigated the feasibility of using tPDT to selectively deplete FAP-expressing CAFs in mice carrying subcutaneous PDAC299 tumors and in *CKP* mice with spontaneously developing PDAC tumors.

The two monoclonal antibodies used in the experiments, 28H1 and negative control DP47GS, could efficiently be functionalized with IRDye700DX. We showed binding of only 28H1-700DX to FAP-overexpressing 3T3 cells in vitro, and cytotoxicity induced by light exposure. Various mechanisms for cytotoxicity by IRDye700DX-conjugated antibodies have been proposed. There is evidence for involvement of ROS-mediated cytotoxicity, as effects can partly be blocked with various ROS scavengers.^35−39^ Photoinduced physicochemical changes of IRDye700DX and subsequent loss of cell membrane integrity was described as another mechanism of cytotoxicity.^40,41^ Most probably, the effects we have observed are the result of a combination of both proposed mechanisms.

We have investigated the biodistribution and therapeutic potential of 28H1-700DX in mice carrying subcutaneous PDAC299 tumors. These tumors show a moderately well-differentiated morphology organized in glandular structures which are typical for adenocarcinomas, and both collagen as well as FAP-expressing cells are abundantly present in the tumor stroma.^42^ The subcutaneous injection of tumor cells leads to highly controlled tumor growth that is easy to monitor, and exposure of the tumor to light can be done superficially through the skin, making this model easier to use than orthotopic models. We found high uptake of both 28H1-700DX and DP47GS-700DX in subcutaneous PDAC299 tumors. While the uptake of 28H1-700DX was heterogeneous throughout the whole tumor, the uptake of DP47GS-700DX was focal and mostly in the periphery. The presence of FAP-expressing fibroblasts throughout the whole tumor as shown with IHC suggests that 28H1-700DX indeed binds to FAP, while the uptake of DP47GS-700DX might be partly FAP-independent. Functionalization of the antibodies with IRDye700DX induces hepatic clearance, and though we would recommend to avoid high substitution ratios to minimize this effect, we do not expect this to hamper clinical translation as the photosensitizer can selectively be activated with light in the tumor regions.^43^

In the therapy experiment, we observed an increase in caspase-3 staining in the PDAC299 tumors that were irradiated compared to non-irradiated tumors in all groups, albeit the most prominent in the 28H1-700DX-treated group. As part of these effects could be explained by heating due to exposure to light,^44^ lower light dose (rates) should be used in follow-up experiments. Surprisingly, we observed autofluorescence in the tumor of PBS-treated mice which was reduced upon exposure to light. We have no explanation for this and it has not been described by others, but it could also add to the upregulation of caspase-3.

As orthotopic models reflect the local environment in which the tumors develop better than subcutaneous models, we have verified our results in the genetically engineered *CKP* mouse model. This model is known to have a fast development of well-differentiated PDAC, making it more suitable for these studies than the slowly progressing models carrying only a KRAS mutation.^45^ As the *CKP* model does not metastasize, future studies into metastasizing potential or abscopal effects could be done in other models such as the *KPC* model.^28^

We observed high uptake of both 28H1-700DX and DP47GS-700DX in the *CKP* tumors at 24 h after injection, though the higher tumor-to-blood ratio for 28H1-700DX suggests specific uptake of this antibody when compared to the DP47GS-700DX control. In the therapy experiments, we did not observe increased caspase-3 expression in the DP47GS-700DX-treated control group, possibly due to the lower light dose used here as compared to the experiment in mice carrying subcutaneous PDAC299 tumours. We found high variation in the induction of apoptosis in the limited number of 28H1-700DX-treated *CKP* mice. This could partly be due to the highly heterogeneous study population, as we did not determine tumor size before inclusion. In a follow-up study, this could be improved through MRI or ultrasound tumor imaging^46^ to assess tumor size before stratifying mice for the treatment. Furthermore, though theoretically 1 h should be sufficient for the upregulation of cleaved caspase-3, effects could be more prominent at 24 h after exposure to light.^47,48^

We have included a limited number of *CKP* mice in these proof-of-principle studies, and in therapy studies the very fast tumor growth could minimize the therapeutic window and complicate follow-up. We recently developed an orthotopically grown model of PDAC299, which leads to slower tumor growth while maintaining the FAP-expressing stromal compartment.^42^ This model is therefore suitable for follow-up studies with 28H1-700DX.

Other groups that have investigated FAP-targeted PDT also indicated the therapeutic potential in preclinical models for breast carcinoma and oesophageal carcinoma.^49−53^ Here, we have shown proof-of-concept of FAP-tPDT in syngeneic PDAC models for the first time. As in most tumor types, including in PDAC, FAP is expressed on cells in the stroma,^54^ we do not expect FAP tPDT to directly kill the tumor cells. Instead, relieving the immune suppressive effects or reducing the interstitial pressure could lead to increased efficacy of existing systemic therapies. The syngeneic models as described here offer the possibility to investigate these biological and immunological effects. Results will always have to be interpreted with caution, as despite promising results of other stromal targeting therapies such as enzymatic targeting of hyaluronic acid^46^ in spontaneous mouse models, the progression free survival was only modestly improved in a subsequent clinical study.^55^ This was in part attributed to the ever existing differences in stroma between PDAC in mice and humans.^3^

If other promising targets on CAFs and/or tumor cells are identified, this approach could easily be modified by conjugating the IRDye700DX to molecules binding those targets. Additionally, various targets on different CAF populations, or targets on CAF and tumor cell populations^56^ could be combined to achieve maximal therapeutic efficacy.

In the current studies, we have added ^111^In to quantify or visualize the uptake and biodistribution in vitro and in preclinical models. A multimodal compound could clinically also be used for preoperative imaging and patient selection (preferably with radionuclides suitable for positron emission tomography such as zirconium-89) or for intraoperative guidance when using a gamma-emitting radionuclide.

## Conclusions

We show feasibility of FAP-tPDT with a monoclonal antibody in syngeneic murine models of subcutaneous and spontaneous PDAC. Future studies in these models could determine the effect of the FAP-tPDT on the tumor microenvironment (e.g., the tissue structure, permeability, and immune cell infiltration) and the synergy of this therapy with other systemic therapies such as chemo- and immunotherapy.

## Supporting Information Available

Overview of animal characteristics, raw data of biodistribution studies, autoradiography and SPECT/CT imaging at 96 h post injection, outlier of DP47GS-700DX in vivo data, in vivo tPDT with 28H1-700DX, in vivo tPDT with DP47GS-700DX, and in vivo tPDT PBS control (PDF)
