# Supplementary material for: Fibroblast Activation Protein-Targeted Photodynamic Therapy of Cancer-Associated Fibroblasts in Murine Models for Pancreatic Ductal Adenocarcinoma
Source: Mol Pharm. 2023 Jul 24;20(8):4319–30. doi: 10.1021/acs.molpharmaceut.3c00453 (PMC10410663; doi:10.1021/acs.molpharmaceut.3c00453)
Supplement: Supplementary file 1 — mp3c00453_si_001.pdf [file mp3c00453_si_001.pdf]

Supplementary data

Fibroblast activation protein-targeted photodynamic therapy of cancer-associated fibroblasts in murine models for pancreatic ductal adenocarcinoma

Daphne N. Dorst[1#], Esther M.M. Smeets[1#], Christian Klein[2], Cathelijne Frielink[1], Daan Geijs[3], Marija Trajkovic-Arsic[4,5], Phyllis F.Y. Cheung[4,5], Martijn W.J. Stommel[6], Martin Gotthardt[1], Jens T. Siveke[4,5], Erik H.J.G. Aarntzen[1], Sanne A.M. van Lith[1*]

[#] Authors contributed equally

[1] Department of Medical Imaging, Radboud University Medical Center, 6525 GA, Nijmegen, The Netherlands

[2] Roche Pharma Research and Early Development, Innovation Center Zurich, 8952 Schlieren, Switzerland

[3] Department of Pathology, Radboud University Medical Center, 6525 GA, Nijmegen, The Netherlands

[4] Bridge Institute of Experimental Tumour therapy, West German Cancer Center, University Hospital Essen, University of Duisburg-Essen, 47057 Essen, Germany

[5] Division of Solid Tumour Translational Oncology, German Cancer Consortium (DKTK Partner Site Essen) and German Cancer Research Center, DKFZ, 69120 Heidelberg, Germany

[6] Department of Surgery, Radboud University Medical Center, 6525 GA, Nijmegen, The Netherlands

*Corresponding author: Sanne A.M. van Lith, Geert Grooteplein zuid 10, 6525 GA, Nijmegen, The Netherlands, sanne.vanlith@radboudumc.nl, 00313613813

**Subcutaneous PDAC299 model**					
Experiment	Tracer (SR IRDye700DX)	Number of animals	Sex	Age (weeks)	
Biodistribution	28H1 (2.74)	14	F	6-8	
Biodistribution	DP47GS (2.28)	10	F	6-8	
PDT	28H1 (2.74)	6	F	6-8	
PDT	DP47GS (2.28)	5	F	6-8	
PDT	PBS	5	F	6-8	
**Spontaneous *CKP* model**					
Experiment	Tracer (SR IRDye700DX)	Number of animals	Sex	Age (weeks)	Time between light exposure and death
Biodistribution	28H1 (1.50 / 2.50)	4	1 M / 3 F	5-6	N.a.
Biodistribution	DP47GS (1.60)	3*	1 M / 2 F	6	N.a.
PDT	28H1 (2.38 / 1.50)	2	1 M / 1 F	6	45 min / 1 hour
PDT	DP47GS (2.29)	3	3 M	6-7	1 hour

Table S1. Overview of the animal characteristics of the mice used in biodistribution and in vivo targeted photodynamic therapy assays. *one mouse was excluded from analysis because of unexplainable extremely high lung uptake. N.a. = not applicable.

	28H1 48 hrs (N=6)	28H1 48 hrs Blocked (N=3)	28H1 96 hrs (N=5)	DP47GS 48 hrs (N=5)	DP47GS 96 hrs (N=5)
Blood	0.58 ± 0.44	0.74 ± 0.07	0.12 ± 0.03	0.68 ± 0.49	0.24 ± 0.31
Muscle	0.35 ± 0.04	0.26 ± 0.05	0.19 ± 0.05	0.31 ± 0.08	0.24 ± 0.07
Tumour	3.30 ± 0.89	2.49 ± 0.27	1.47 ± 0.21	3.10 ± 0.36	2.65 ± 1.17
Heart	1.40 ± 0.11	1.60 ± 0.15	0.77 ± 0.21	1.80 ± 0.25	1.21 ± 0.46
Lung	0.88 ± 0.14	1.10 ± 0.01	0.42 ± 0.13	1.12 ± 0.25	0.61 ± 0.28
Liver	41.46 ± 4.71	41.77 ± 2.82	27.57 ± 7.10	54.57 ± 13.25	38.78 ± 7.46
Spleen	9.14 ± 0.92	9.11 ± 0.17	5.18 ± 1.82	12.67 ± 3.32	8.60 ± 2.51
Pancreas	1.86 ± 2.31	0.48 ± 0.08	0.33 ± 0.11	0.70 ± 0.21	0.55 ± 0.30
Stomach	0.73 ± 0.11	0.55 ± 0.02	0.37 ± 0.08	0.90 ± 0.31	0.44 ± 0.16
Duodenum	1.50 ± 0.31	1.35 ± 0.23	0.68 ± 0.19	1.10 ± 0.48	0.67 ± 0.36
Tibia + bone marrow	5.41 ± 0.75	4.05 ± 0.38	3.10 ± 0.52	5.92 ± 1.58	3.66 ± 1.72
Kidney	1.50 ± 0.10	1.41 ± 0.06	1.00 ± 0.18	2.90 ± 0.80	1.85 ± 0.42

Table S2. Raw data of biodistribution of ^{111}In-labelled 28H1-700DX and DP47GS-700DX at 48 or 96 hours post intravenous injection in mice carrying subcutaneous PDAC299 tumours, depicted as percentage of injected activity dose per gram of tissue (%IA/g).

	28H1 24 hrs (N=4)	DP47GS 24 hrs (N=2)
Blood	8.22 ± 4.17	25.85 ± 1.10
Muscle	2.28 ± 1.25	1.50 ± 2.12
Tumour	13.12 ± 3.55	14.66 ± 2.25
Heart	4.20 ± 1.18	11.47 ± 0.86
Lung	5.07 ± 1.17	5.43 ± 0.79
Liver	15.86 ± 4.23	33.22 ± 0.77
Spleen	4.49 ± 3.43	18.62 ± 1.57
Pancreas	9.31*	9.67*
Stomach	2.59 ± 0.84	6.22 ± 1.36
Duodenum	4.51 ± 2.70	7.44 ± 7.49
Tibia + bone marrow	12.70 ± 2.38	7.20 ± 3.70
Kidney	4.95 ± 1.49	15.75 ± 0.80

Table S3. Raw data of biodistribution of ^{111}In-labelled 28H1-IRDye700DX and DP47GS-700DX at 24 hours post intravenous injection in CKP mice carrying spontaneous pancreatic tumours, depicted as percentage of injected activity dose per gram of tissue (%IA/g). * only one mouse per group had normal pancreatic tissue left, in the other mice only tumour tissue was found.

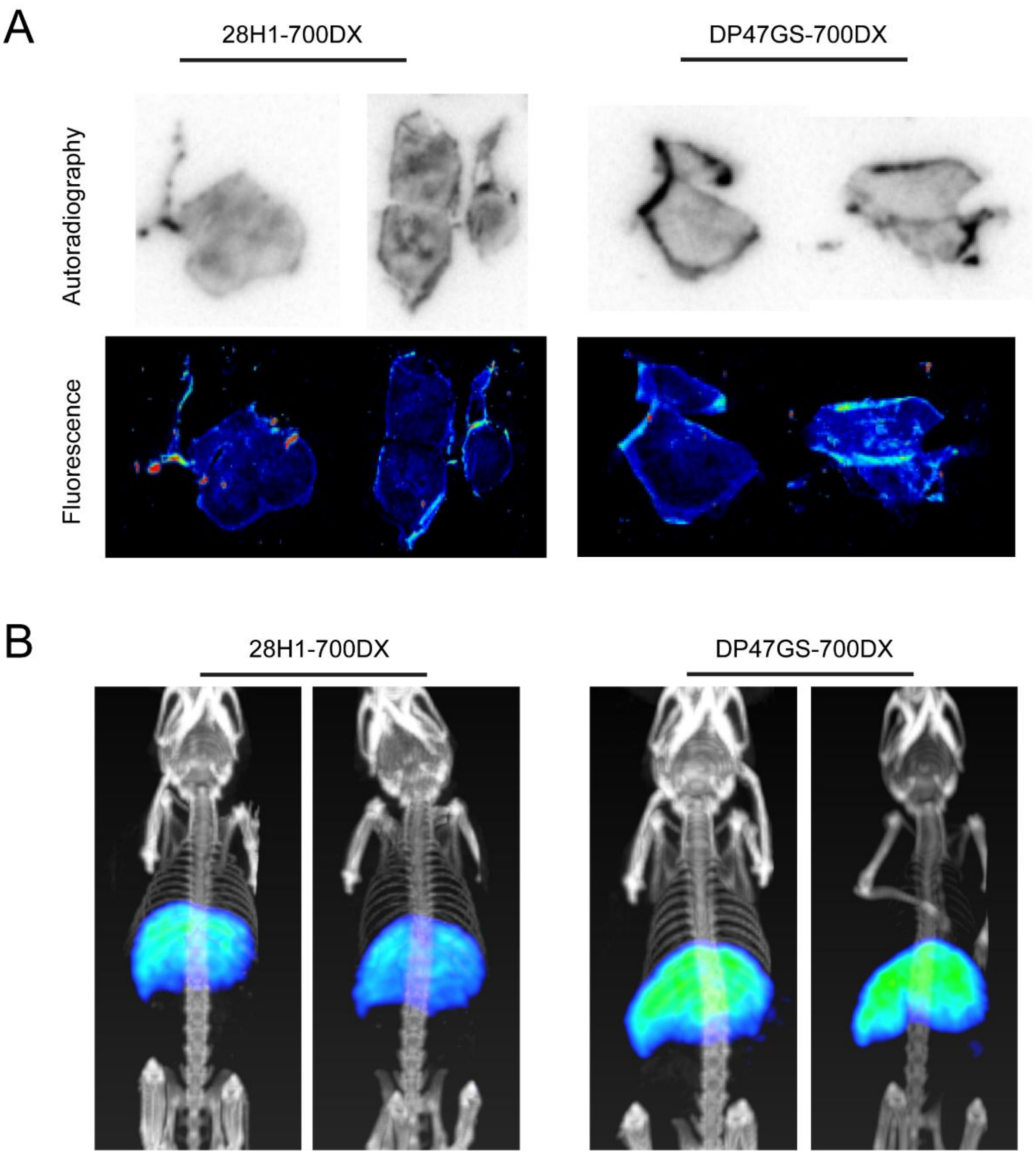

Figure S1. A) Autoradiography and fluorescence imaging of tumour tissue sections at 96 hours post injection of 10 MBq ^{111}In-labelled 28H1-700DX or DP47GS-700DX. B) SPECT/CT scan of mice carrying subcutaneous PDAC299 tumours at 96 hours post injection of 10 MBq ^{111}In-labelled 28H1-700DX or DP47GS-700DX. Note that only the uptake in the liver is visualized.

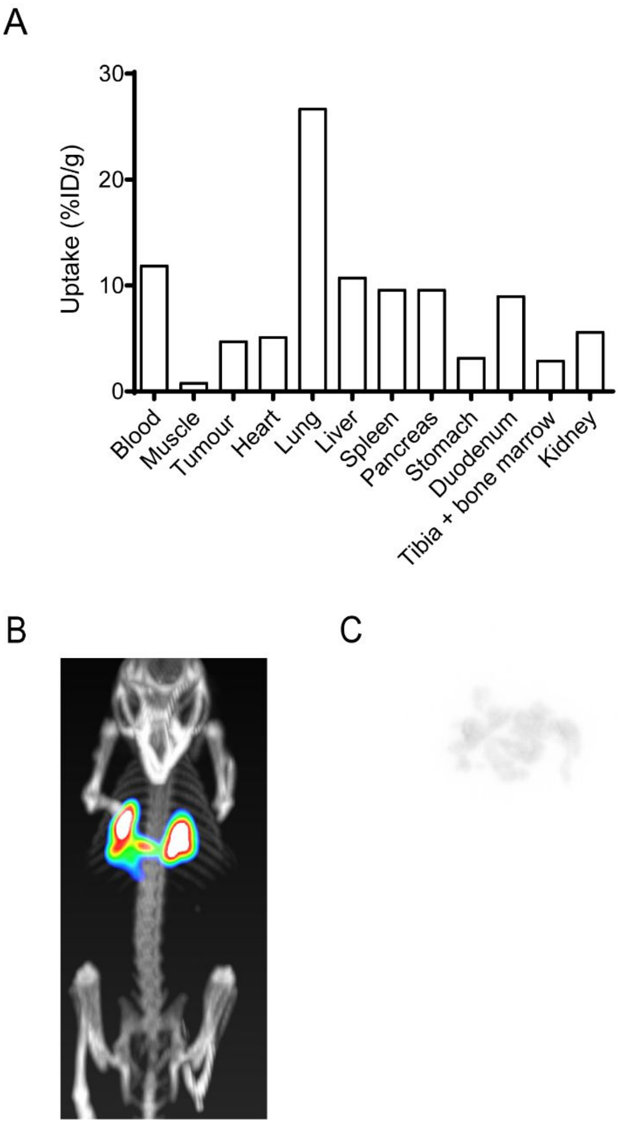

Figure S2. Outlier in the in vivo evaluation of DP47GS-700DX in a CKP mouse, showing A) uptake in various tissues at 24 hours after injection of ^{111}In-labelled DP47GS-700DX, depicted as percentage of injected dose per gram (%ID/g) B) SPECT/CT scan at 24 hours post injection of 10 MBq ^{111}In-labelled DP47GS-700DX C) autoradiography of a tumour section at 24 hours post injection of 10 MBq ^{111}In-labelled DP47GS-700DX. Note that the lung uptake is very high and that no tumour uptake is visualized on SPECT/CT or in autoradiography.

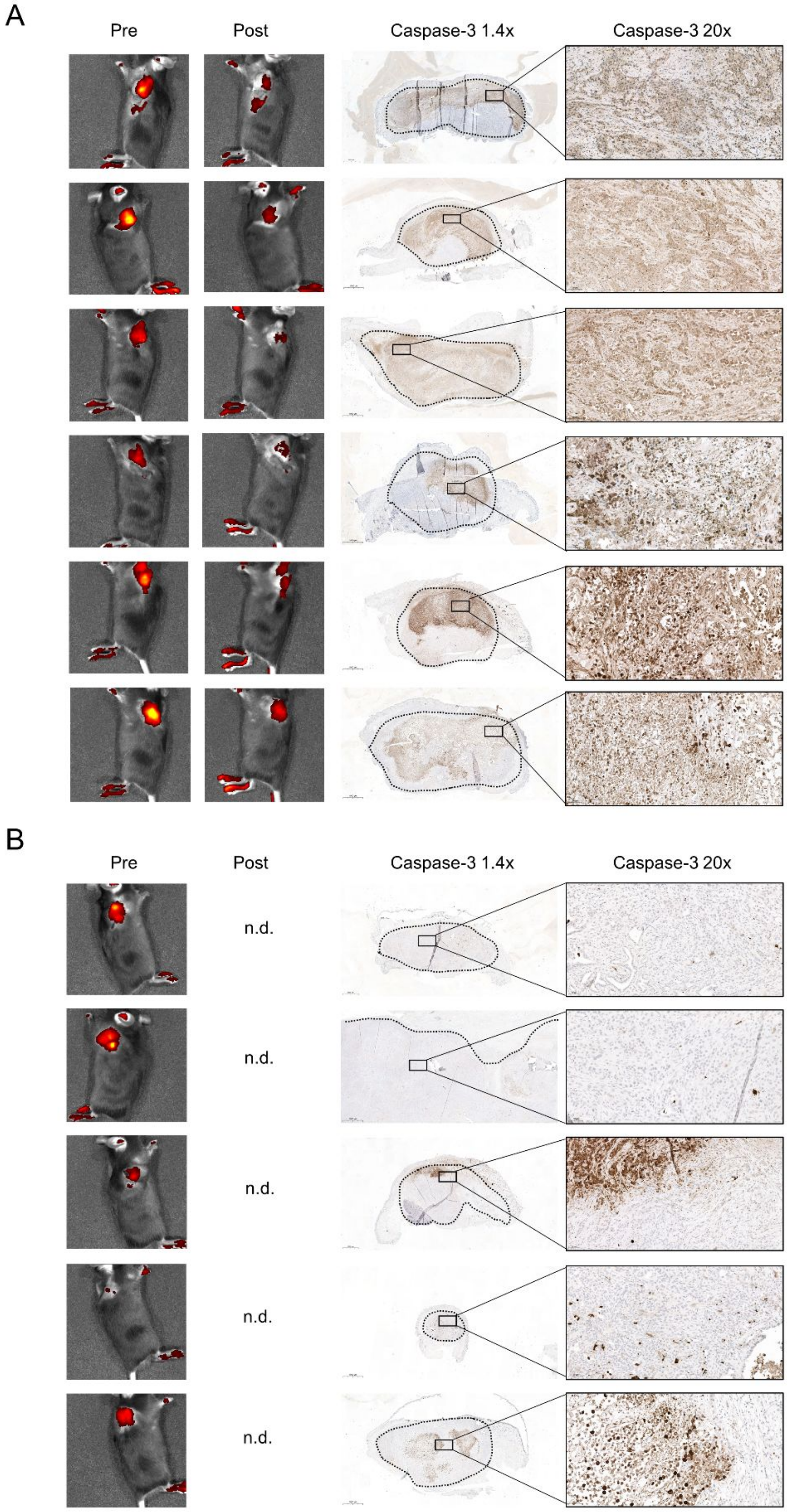
A
Pre
Post
Caspase-3 1.4x
Caspase-3 20x
B
Pre
Post
Caspase-3 1.4x
Caspase-3 20x
n.d.
n.d.
n.d.
n.d.
n.d.

Figure S3. In vivo tPDT with 28H1-700DX. Mice carrying two subcutaneous PDAC299 tumours were injected with 50 µg 28H1-700DX. 48 hours after injection fluorescence was visualized (pre), then one of the tumours was exposed to 100 J/cm^2 230 mW/cm^2 of 690 nm light and fluorescence was visualized again (post). Tumours were formalin fixed and paraffin embedded and induction of apoptosis was assessed with cleaved-caspase-3 IHC, of which a 1.4x and 20x magnification is shown. Of note, one mouse developed only one tumour which was added to the irradiated group.

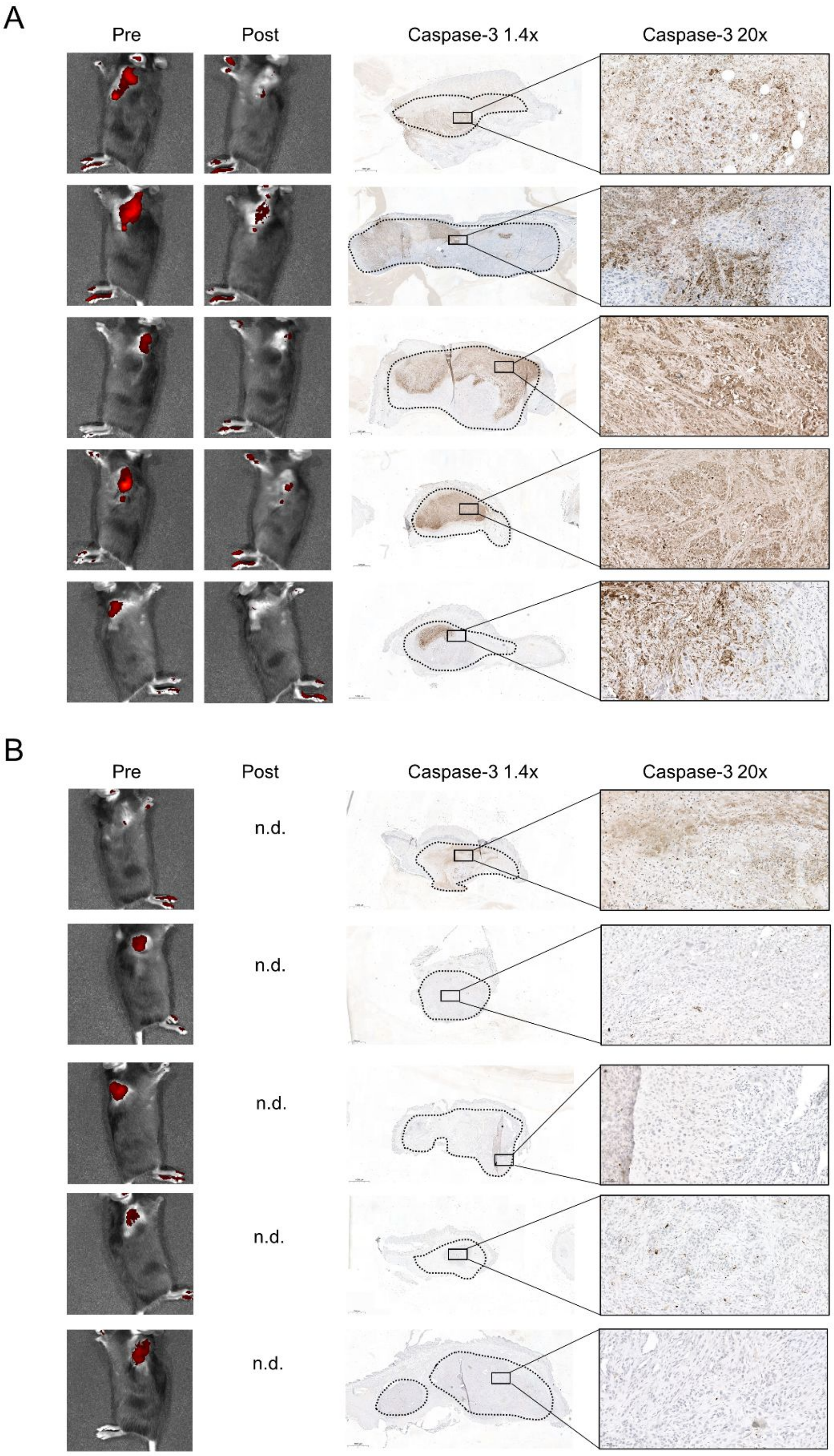

Figure S4. In vivo tPDT with DP47GS-700DX. Mice carrying two subcutaneous PDAC299 tumours were injected with 50 µg DP47GS-700DX. 48 hours after injection fluorescence was visualized (pre),

then one of the tumours was exposed to 100 J/cm^2 230 mW/cm^2 of 690 nm light and fluorescence was visualized again (post). Tumours were formalin fixed and paraffin embedded and induction of apoptosis was assessed with cleaved-caspase-3 IHC, of which a 1.4x and 20x magnification is shown.

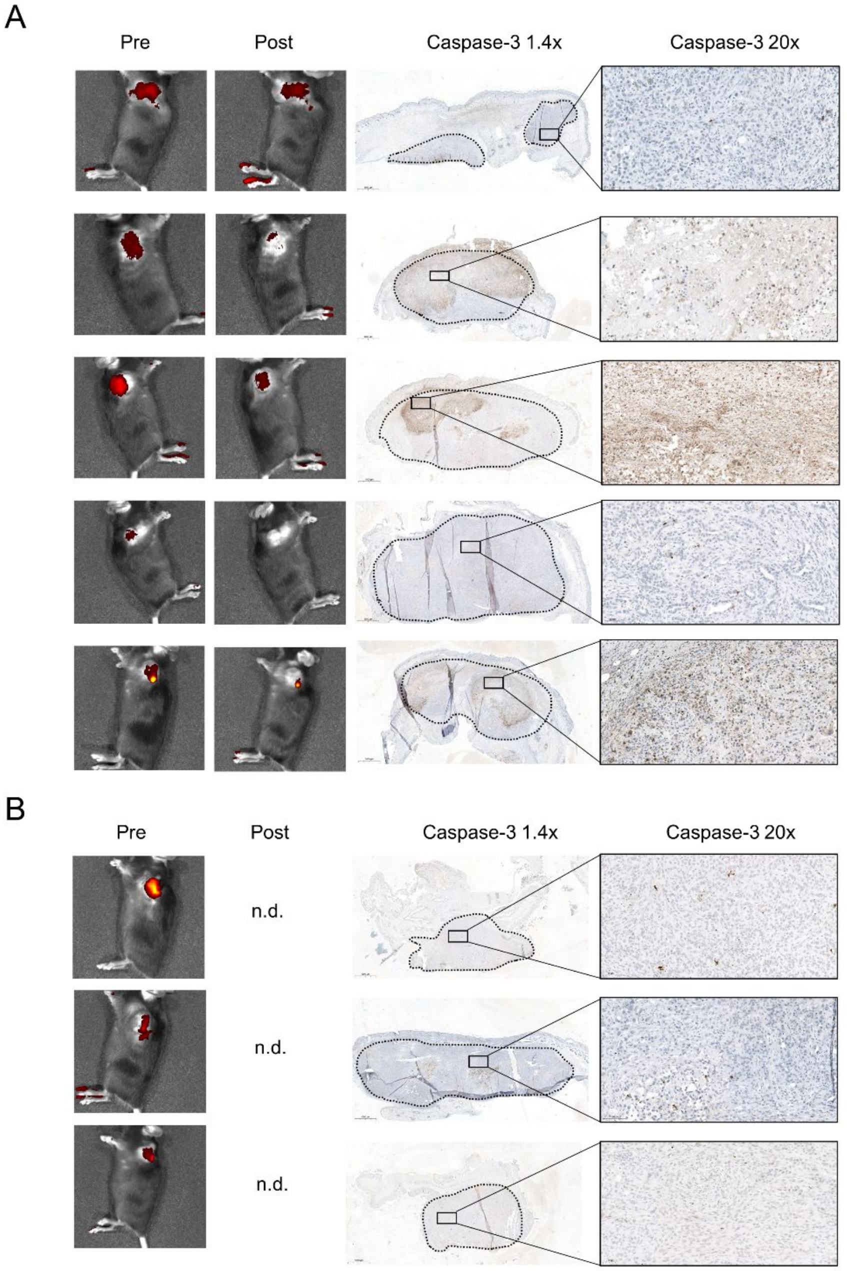

Figure S5. PBS control group for in vivo tPDT. Mice carrying two subcutaneous PDAC299 tumours were injected with PBS. 48 hours after injection fluorescence was visualized (pre), then one of the tumours was exposed to 100 J/cm^2 230 mW/cm^2 of 690 nm light and fluorescence was visualized again (post). Tumours were formalin fixed and paraffin embedded and induction of apoptosis was assessed with cleaved-caspase-3 IHC, of which a 1.4x and 20x magnification is shown. Of note, two mice developed only one tumour, which were added to the irradiated group.
